# Assessment of EHR Efficiency Tools and Resources Associated with Physician Time Spent on the Inbox

**DOI:** 10.1007/s11606-024-08761-3

**Published:** 2024-05-08

**Authors:** Richa Bundy, Adam Moses, Elisabeth Stambaugh, Paschal Stewart, Lauren Witek, Lindsey Carlasare, Gary Rosenthal, Christine Sinsky, Ajay Dharod

**Affiliations:** 1 Informatics and Analytics (I&A), Department of Internal Medicine, Wake Forest School of Medicine, Winston-Salem, USA; 2 Wake Forest Health Network, Atrium Health Wake Forest Baptist, Winston-Salem, NC USA; 3 Wake Forest Center for Biomedical Informatics (WFBMI), Wake Forest School of Medicine, Winston-Salem, USA; 4 Professional Satisfaction and Practice Sustainability, American Medical Association, Chicago, IL USA; 5 General Internal Medicine (GIM), Department of Internal Medicine, Wake Forest School of Medicine, Winston-Salem, USA; 6 Department of Implementation Science (IS), Wake Forest School of Medicine, Division of Public Health Sciences (PHS), Winston-Salem, USA; 7 Wake Forest Center for Healthcare Innovation (CHI), Wake Forest School of Medicine, Winston-Salem, USA

**Keywords:** inbox time, EHR inbox efficiency, primary care, EHR use metrics

## Abstract

**Background:**

Physicians are experiencing an increasing burden of messaging within the electronic health record (EHR) inbox. Studies have called for the implementation of tools and resources to mitigate this burden, but few studies have evaluated how these interventions impact time spent on inbox activities.

**Objective:**

Explore the association between existing EHR efficiency tools and clinical resources on primary care physician (PCP) inbox time.

**Design:**

Retrospective, cross-sectional study of inbox time among PCPs in network clinics affiliated with an academic health system.

**Participants:**

One hundred fifteen community-based PCPs.

**Main Measures:**

Inbox time, in hours, normalized to eight physician scheduled hours (IB-Time_8_).

**Key Results:**

Following adjustment for physician sex as well as panel size, age, and morbidity, we observed no significant differences in inbox time for physicians with and without message triage, custom inbox QuickActions, encounter specialists, and message pools. Moreover, IB-Time_8_ increased by 0.01 inbox hours per eight scheduled hours for each additional staff member resource in a physician’s practice (*p* = 0.03).

**Conclusions:**

Physician inbox time was not associated with existing EHR efficiency tools evaluated in this study. Yet, there may be a slight increase in inbox time among physicians in practices with larger teams.

## INTRODUCTION

Physicians spend a substantial portion of their workday in the electronic health record (EHR). Over 20% of a physician’s time in the EHR can be attributed to management of their inbox.^1^ A physician’s inbox is treated as a catch-all application for messages from patients and staff as well as automated alerts and notifications from the EHR. Furthermore, the volume of inbox messages has risen concurrently with the rate of patient messages following the COVID-19 pandemic, with rates reported between 33 and 49 messages per day.^2,3^ This rising EHR burden has been linked to physician burnout, turnover, and increased physiological stress.^3–6^ Previous studies have demonstrated physician dissatisfaction with the time and burden spent on administrative tasks in the inbox^3^. In attempts to reduce inbox burden and associated burnout, suggestions have been made to optimize physician time and efficiency by utilizing inbox collaboration tools such as message pools, message triage, and shared inboxes.^7,8^ Additionally, EHRs that offer customizable inbox actions to streamline repetitive inbox workflows may offer improved efficiency by reducing mouse-clicks.^9,10^ The time on inbox metric, or IB-Time_8_, was developed as a standardized way to measure physician inbox time and effort burden. IB-Time_8_ tracks the amount of time spent in the EHR inbox for every 8 h of scheduled patient appointments.^11^ This study explores the association between various existing EHR efficiency tools and clinical staff resources with IB-Time_8_ among primary care physicians.

## METHODS

### Setting

This retrospective, cross-sectional study was conducted at a large academic medical health system. We included primary care physicians whose primary department was within one of the organization’s network clinics.

### Data

The main outcome was a standardized time on inbox measure, IB-Time_8_. This standardized metric measures the time clinicians spend on the inbox per eight scheduled hours of patient visits. We selected this metric as it aligns with our objective to employ a well-established, standardized, and normalized measure, facilitating consistent derivation and scaling across various health systems. We aggregated clinician inbox time and schedule data to calculate IB-Time_8_ using audit log data available in the electronic health record (EHR) data warehouse between July 1, 2021, and June 30, 2022. The audit log data contains hourly activity in seconds for users in the EHR. We limited the audit log data to activities associated with the inbox and aggregated the total time per physician per day. To validate that the list of audit log inbox activities was comprehensive, we systematically sent and received messages in the EHR test environment and extracted the subsequent audit log activities. Furthermore, we compared the EHR vendor-derived inbox time metrics to our calculated inbox times using a paired *t*-test to ensure concordance. We also extracted the number of scheduled patient visit hours per physician per day according to EHR visit data. We then calculated the average IB-Time_8_ metric for each physician by dividing the total time on the inbox by the total number of scheduled hours and multiplying by eight.

Predictors of inbox time were determined by the authors a priori. Data elements were collected from the EHR or reported by administrative leaders. For instance, the EHR records whether a physician was included in an inbox message pool or had set up a custom inbox QuickAction. A message pool is a shared inbox that allows clinician team members to respond rather than the physician. QuickActions are macro-like functions within the inbox that allow users to perform common tasks that would otherwise take several steps. QuickAction and message pool information were collected as a snapshot at a single time point following the study period. Data elements including clinic use of inbox message triage, physician use of encounter specialists, and the number of non-physician medical staff at each clinic were reported by clinic managers using a templated data collection form. This data was collated and validated by the physician network Chief Medical Officer (CMO). Encounter specialists are certified medical assistants (CMAs) who function within a Patient-Centered Medical Home (PCMH) and guide patients through their clinic encounter. Encounter specialists perform all basic CMA functions with an increased focus on ensuring quality care is delivered; they also may serve as medical scribes for physicians.

We also extracted EHR data on physician sex, age, panel size, percentage of panel over age 55, and percentage of panel with Charlson Comorbidity Index (CCI) more than two.^12^ We designated percentage of panel over age 55 and CCI more than two as proxies for panel complexity. Patients were attributed to physicians based on which physician they saw most often in the prior year, or the most recently visited physician if multiple physicians were seen equally as often. We explored the association between IB-Time_8_ and these physician and patient panel characteristics.

### Analysis

All data elements were aggregated by physician into one dataset with outcome, predictor, and covariate variables. We assessed the association between average IB-Time_8_ and the various predictors using simple bivariate comparisons as well as adjusted regression analyses. We used linear mixed effects regression to account for within-clinic correlation, as physicians in the same clinic may have more similar resources and patient populations. Regression models were adjusted for physician sex, inbox message volume, panel size, percentage of panel greater than age 55, and percentage of panel with CCI greater than two. Model covariates were selected a priori based on available data and clinical judgment. Observations with missing data were excluded from the analyses. All analyses were performed using R software, version 4.1.1. This study was approved by the institutional review board of Wake Forest University School of Medicine (IRB00079182).

## RESULTS

We included 115 physicians from 48 different clinics in this analysis. Descriptive statistics on the population and predictors are illustrated in Table 1. On average, physicians were 50 years of age and 53% were male. The average physician panel size was 1140 patients with an average of 54% of the panel over the age of 55 and 16% medically complex according to their CCI. Most physicians worked in clinics with message triage (91%) or had at least one custom QuickAction (87%) set up in their inbox. Conversely, message pools (38%) and encounter specialists (24%) were less common among physicians. The average physician spent 0.66 h, or 40 min, in the inbox per 8-h scheduled day.

**Table 1:** Physician Characteristics

	**Total physicians**
	**(*N* = 115)**
Physician age, mean (SD)	50.3 (12.3)
Physician sex	
Female	53 (46.1%)
Male	62 (53.9%)
Physician panel size, mean (SD)	1140 (502)
Physician panel age > 55 (%), mean (SD)	53.7 (23.3)
Physician panel CCI > 2 (%), mean (SD)	15.9 (10.1)
IB messages per day, mean (SD)	33.3 (13.9)
Physician resources	
Staff resources, mean (SD)	10.0 (5.43)
Physician in IB message pool	44 (38.3%)
Physician has custom IB QuickAction	100 (87.0%)
Staff performs message triage	105 (91.3%)
Physician utilizes encounter specialist	28 (24.3%)
IB-Time_8_, mean (SD)	0.66 (0.29)

**Figure 1:**
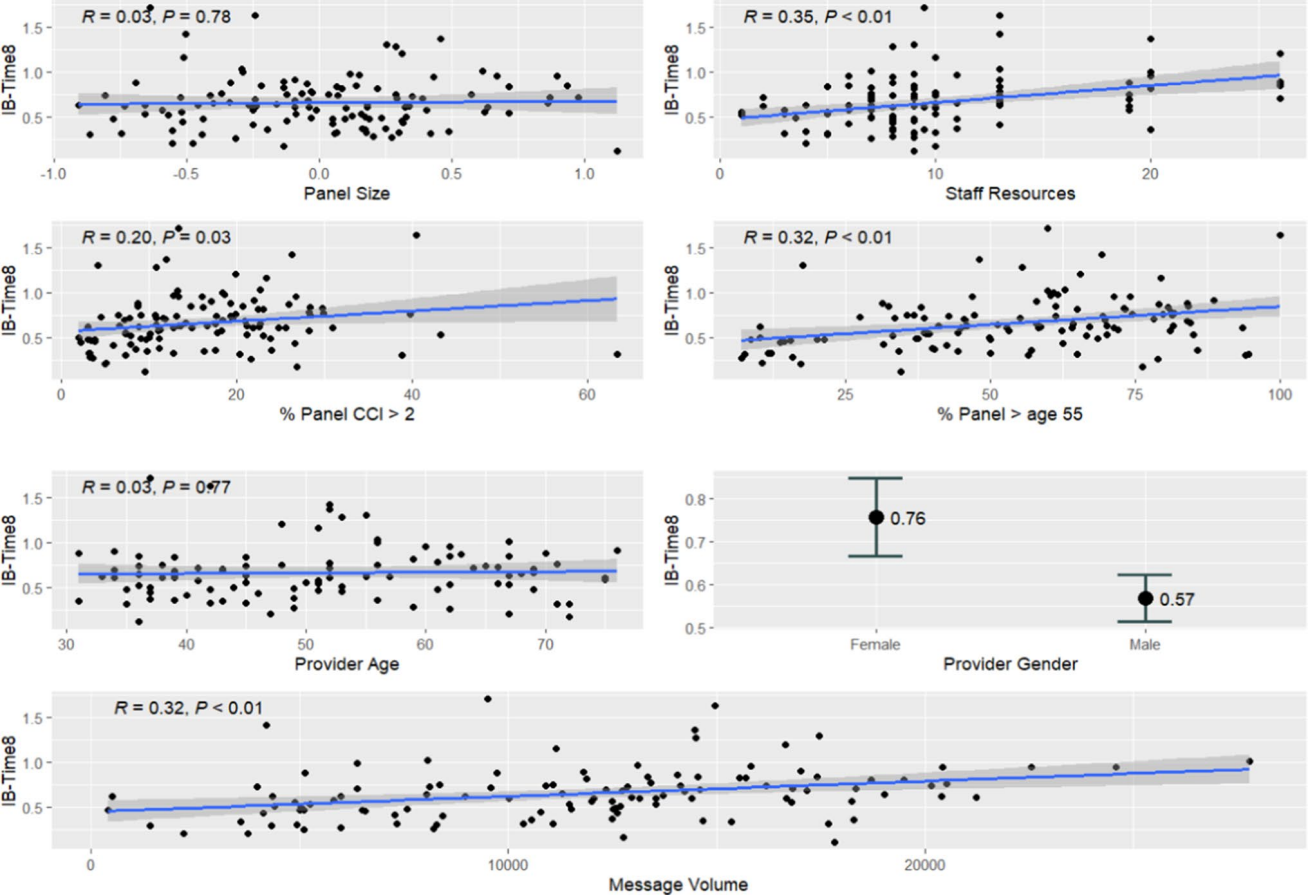
IB-Time_8_ by physician, clinic, and patient panel characteristics.

Associations between IB-Time_8_ and physician attributes are displayed in Fig. 1. Female physicians spent an average of 0.19 h (11.36 min) longer in the inbox than male physicians (95% CI, 5.03–17.68 min, *p* < 0.001), per eight scheduled hours. Additionally, IB-Time_8_ had a positive correlation with percent of panel over 55 (Pearson’s *r* = 0.32 [95% CI, 0.15 to 0.49]; *p* < 0.001), percent of panel with CCI more than 2 (Pearson’s *r* = 0.20 [95% CI, 0.02 to 0.37]; *p* = 0.03), and message volume (Pearson’s *r* = 32 [95% CI, 0.14 to 0.47]; *p* < 0.001). However, panel size (Pearson’s *r* = 0.03 [CI, − 0.16 to 0.21]; *p* = 0.78) and physician age (Pearson’s *r* = 0.03 [CI, − 0.17 to 0.22]; *p* = 0.77) were not significantly correlated with IB-Time_8_.

**Figure 2:**
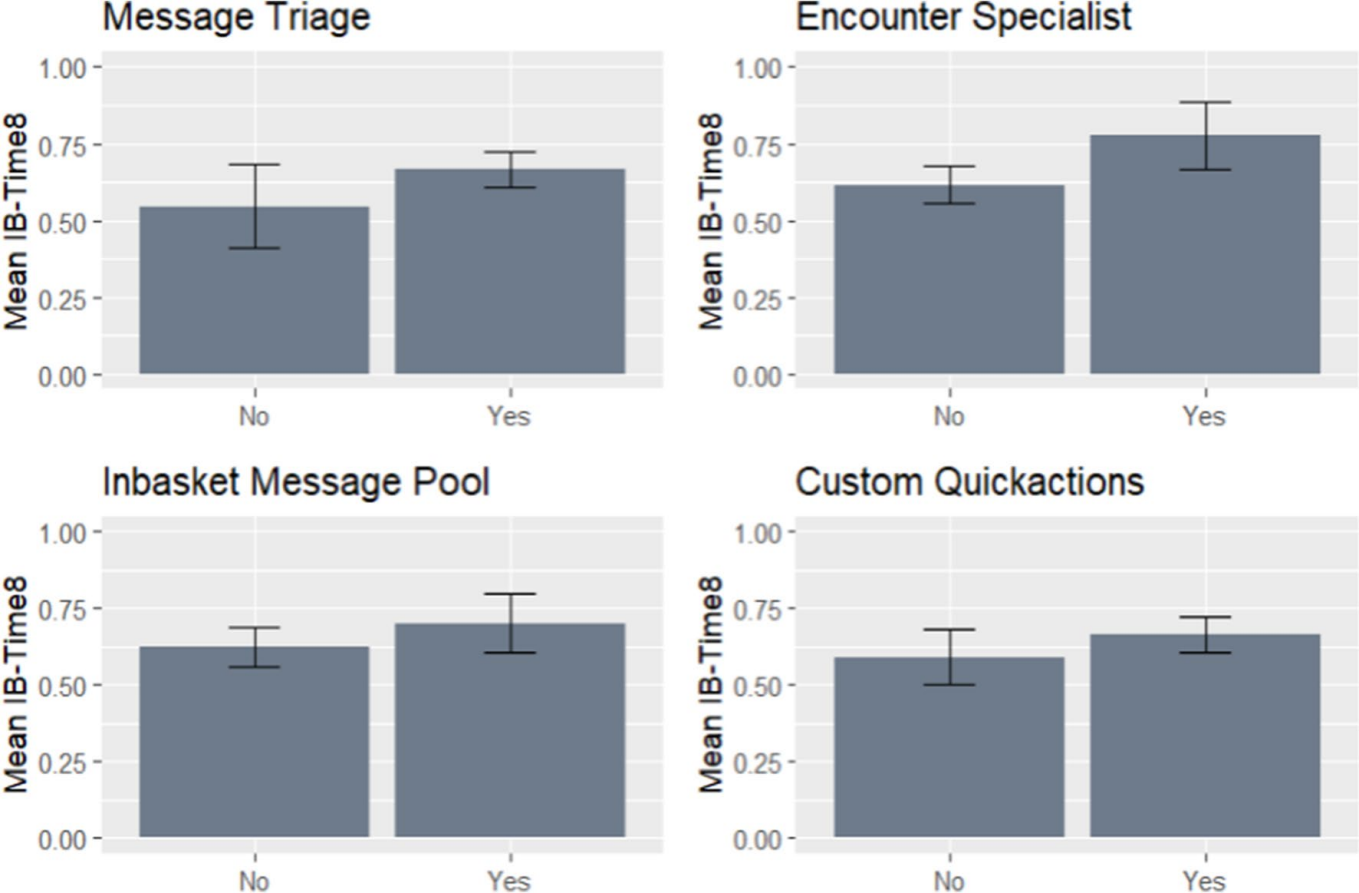
Unadjusted mean IB-Time_8_ by physician adoption of EHR efficiency tool or resource.

In unadjusted, bivariate analysis, we did not find significant associations between inbox time and custom QuickActions, message pools, or message triage (Fig. 2). Conversely, we found that physicians working in clinics with the encounter specialist model or in clinics with a higher number of clinical staff resources had higher IB-Time_8_. These associations remained significant after adjusting for physician sex, panel size, message volume, and panel complexity attributes (Table 2). On the other hand, we did not observe statistically significant differences in IB-Time_8_ among physicians with custom EHR QuickActions, message pools, or message triage workflows in adjusted analyses.

**Table 2:** Differences in IB-Time_8_ by Clinic Resource

Predictor	No. (%) of physicians with predictor	Mean (SD) IB-Time_8_ among physicians with predictor	Mean (SD) IB-Time_8_ among physicians without predictor	Mean difference in IB-Time_8_ (95% CI)	*p*-value	Regression adjusted difference in IB-Time_8_^*^ (95% CI)	*p*-value
Encounter specialist	28 (24.35%)	0.78 (0.28)	0.62 (0.28)	0.16 (0.04 to 0.29)	0.01	0.10 (− 0.03 to 0.22)	0.15
Message pool	44 (38.26%)	0.70 (0.32)	0.63 (0.27)	0.07 (− 0.04 to 0.19)	0.18	0.04 (− 0.05 to 0.14)	0.52
Custom QuickAction	100 (87.00%)	0.66 (0.30)	0.59 (0.16)	0.07 (− 0.03 to 0.18)	0.78	0.07 (− 0.06 to 0.21)	0.28
Message triage	105 (91.30%)	0.67 (0.29)	0.54 (0.19)	0.13 (− 0.02 to 0.27)	0.30	0.06 (− 0.13 to 0.26)	0.52
Total staff resources^†^	--	--	--	0.02 (0.01 to 0.20)	0.003	0.01 (0.00 to 0.02)	0.04

^*^Adjusted for panel size, percent panel greater than 55, percent panel with CCI > 2, message volume, and provider sex

^**†**^Difference in IB-Time_8_ with each additional staff resource, in inbox hours per eight scheduled hours

## DISCUSSION

This study sought to determine whether inbox efficiency tools and resources were associated with reduced inbox time among primary care physicians in our network clinics. The data did not suggest that presence of inbox efficiency tools and staff resources led to lower physician time on the inbox. Contrarily, we found inbox time was higher among physicians with access to encounter specialists and additional staff resources. Furthermore, those who set up message pools, message triage workflows, and custom QuickActions spent a similar amount of time on inbox work as those who did not utilize these efficiency tools.

Prior literature has discussed tactics for reducing the inbox burden among physicians including collaborating and delegating messages to non-physician staff by implementing message pools and message triage workflows.^9,13–15^ Yet, only a couple quantitative studies exist on how these types of interventions impact inbox time. Fogg and Sinsky reported reduced volume of patient messages following a robust intervention focused on triaging patient portal messages. However, this study only accounted for message volume and not time on the inbox.^7^ In addition, Nguyen et al. assessed numerous EHR proficiency tools including QuickActions, and found no significant associations with inbox time.^16^ Similarly, the present study also observed no differences in inbox time between physicians with and without message triage, message pools, and custom QuickActions. Alternatively, we observed higher inbox time among physicians who had access to team support through resources such as encounter specialists and additional non-physician staff.

There are potential explanations for our counterintuitive findings. We did not have data on dates of implementation of efficiency tools and resources and therefore could not perform pre- versus post-intervention analyses. We could be observing reverse causation bias, in which physicians with higher inbox burden were more likely to seek out additional tools, set up triage workflows, and onboard additional staff resources. Another possibility is physicians with efficiency tools dedicated the same time to inbox work but were able to tackle more complex messages in this time. However, we did not quantify message complexity in this study. The finding that encounter specialists and additional staff resources contribute to more inbox time is also unexpected. Encounter specialists do not primarily focus on inbox work and therefore may be more likely to impact other aspects of physician efficiency in the EHR. In supplemental analyses, we found those with encounter specialists spent 53 min less on total EHR time per eight scheduled hours (EHR-Time_8_) in unadjusted analyses (*p* = 0.04); however, this finding failed to remain significant after adjustment for physician gender, panel size, message volume, and panel complexity (*p* = 0.06). Additional staff resources were also associated with higher inbox time, which is surprising, as they could theoretically be utilized to help with inbox management. It is possible that physicians with more clinic staff did not have the necessary workflows in place to help support the inbox. Additionally, larger teams may be more likely to rely on the EHR for communication rather than face-to-face, which may explain our observed increase in IB-time_8_ as staff resources increase. Another potential explanation for our counterintuitive findings is that encounter specialists and clinic staff may have contributed to inbox-related or inbox-derived work not captured by audit log data, such as calling patients to schedule visits in response to a patient message, which then allowed physicians to spend more time on the inbox.

This study has notable strengths. We used a widely accepted, normalized metric to measure inbox time using data from an EHR relational database. This metric can be adapted to other health systems with a similar EHR data structure. Also, the inbox metric was developed using audit log data and is therefore not as susceptible to measurement changes as it would if developed using vendor-derived metrics. Additionally, the use of community practice physician data adds to the generalizability of our findings. Physicians in these clinics are also likely to work at one clinic, limiting potential bias that would arise if we included physicians who split their time between clinics with varying inbox workflows and resources. Furthermore, our analysis is adjusted for important confounders tied to inbox time such as physician sex, panel size, panel morbidity, and panel age.^17,18^

We also observed limitations to this study. As a retrospective and cross-sectional study, we were only able to explore associations, and precluded from establishing causal relationships. We had a limited sample size of physicians within a single health system. We are also limited by how the audit log data tracks time in the inbox. The EHR only logs time spent on inbox activities and does not capture time spent on inbox-related activities such as viewing results or notes. Additionally, the EHR stops recording UAL time after 5 s of mouse or keystroke inactivity.^19^ Therefore, we estimate that the inbox time presented in the study is underestimating the true amount of time physicians spend on inbox-related work. For instance, prior research using stop-motion analysis of EHR time found physicians spent an average of 84 min per calendar day on inbox activities, compared to the 40 min per eight scheduled hours reported in this study.^1^ Regardless, these metric limitations are consistent throughout the sample of physicians equally. Therefore, we do not believe this limitation impacted the primary objective of this study.

We also encountered limitations due to the timing of data collection on QuickActions, staff resources, and message pools in comparison to the study period. These resources may have been added during or after the study period and led to misclassification bias of physician efficiency tools and resources. In addition, we acknowledge some of the predictors of inbox time were more directly tied to inbox work than others. For example, inbox custom QuickActions should directly impact the efficiency of physicians in the inbox, while encounter specialists are less likely to focus on inbox work. Physicians with encounter specialists were also observed to have larger panels and more complex patients, although not statistically significant. However, when we assessed encounter specialists’ impact on other EHR time metrics such as total EHR time, we did not find significant differences after adjusting for physician sex, panel size, and panel complexity. Furthermore, we were unable to ascertain data on the extent to which QuickActions were used by physicians. The presence of a custom QuickAction is not indicative of the use of that functionality on a regular basis, which may explain why we were unable to detect any inbox efficiencies associated with these tools. Furthermore, achieving EHR efficiency may depend on the level of training and experience with these tools, which was not assessed in this study. Additionally, there are other patient factors we could have considered such as patient sex. Namely, Rittenberg et al. demonstrated that female physicians with a higher proportion of males in their panel spent more time on their inbox.^18^ Therefore, adjusting for both physician sex and patient sex may have been a worthwhile consideration not evaluated in the present study.

In summary, we did not find data to support the hypothesis that message pools, custom QuickActions, and message triage contribute to lower inbox time among primary care physicians working in community practices. Furthermore, we found that larger clinical teams and the presence of encounter specialists contributed to higher inbox time. The present study is one of few that quantitatively assesses the impact of interventions aimed at improving inbox efficiency and reducing time spent on the inbox. By challenging our hypothesis, the study highlights the complexity of physician workload. Our findings underscore the need for additional quantitative, qualitative, and mixed-method studies to draw conclusions on types of inbox tools that may effectively reduce inbox time. Future studies with prospective implementations should also examine the best practices for using tools such as those outlined in this study or additional interventions such as ambient listening documentation software, eliminating certain automated inbox messages, and billing for patient messages. Additionally, potential analyses could also focus on broader outcomes, such as responsiveness to patient inquiries, quality of care, health outcomes, and message complexity to more comprehensively evaluate EHR efficiency tools.
